# Supplementary material for: MetaRibo-Seq measures translation in microbiomes
Source: Nat Commun. 2020 Jun 29;11:3268. doi: 10.1038/s41467-020-17081-z (PMC7324362; doi:10.1038/s41467-020-17081-z)
Supplement: Supplementary file 10 — Supplementary Data 7 [file 41467_2020_17081_MOESM10_ESM.zip › File2/Confidence_High_Taxonomy/387378_out.krona.html]

Javascript must be enabled to view this page.

members
magnitude
magnitudeUnassigned
count
unassigned
taxon
rank

387378\_out

4

SRS023914\_contig\_number\_2733SRS053356\_contig\_number\_35136SRS098571\_contig\_number\_65196SRS1041145\_contig\_number\_contig-100\_1557.173214
5

2
superkingdom
1

201174
phylum
1

1760
1
class

order
1
85006

1
family
1268

1
genus
1742989


SRS013687\_contig\_number\_20366
1933880
species
1
